# Asthma as a child: continuous attentiveness to breathing to participate in everyday activities – need for support

**DOI:** 10.1177/17449871241264742

**Published:** 2024-09-27

**Authors:** Hildegunn Sundal

**Affiliations:** Associate Professor, Molde University College, Molde, Norway

**Keywords:** asthma, children, everyday activities, hermeneutic phenomenological, in-depth interviews

## Abstract

**Background:**

Asthma is one of the most common chronic diseases in childhood.

**Aims:**

This study explores the experiences of participating in everyday activities among children with asthma.

**Methods:**

The study employed a qualitative design and was grounded in a hermeneutic-phenomenological approach. The data were collected through in-depth interviews with four children aged 9–12 diagnosed with asthma.

**Results:**

One theme was ‘heavy breathing stopping the body in motion’, and subthemes were as follows: ‘experiencing intrusive wheezing’, ‘calming one’s breath to participate’ and ‘being sick and refraining from participating’. Another theme was ‘help in everyday life and support to participate’, and subthemes were as follows: ‘taking the necessary asthma medicine’, ‘being understood and met as sick’ and ‘adapting one’s leisure activities’.

**Conclusions:**

To participate in everyday activities, children with asthma require insight into and an understanding of the importance of taking asthma medicine as recommended. They also need help in everyday life and support to participate in everyday activities. Children taking care of their own illnesses and listening to their bodies provide them with the greatest degree of participation in activities in their everyday lives. These findings represent an important contribution to the understanding of nursing care for children with asthma.

## Introduction

Asthma is one of the most common chronic diseases in childhood (Anandan et al., 2010; Serebrisky and Wiznia, 2019), and thus, the key focus of this paper is children’s participation in everyday activities when they have asthma.

Children’s experiences of living with asthma can be understood through their descriptions of the symptoms associated with asthma. However, a review of 21 studies (Wallace-Farquharson et al., 2022) described children’s and adolescents’ descriptions of asthma symptoms as limited. Specifically, children and adolescents tend to describe living with asthma using standard terminology related to asthma symptoms, such as cough, wheeze, chest tightness and shortness of breath. Furthermore, children and adolescents also use nonstandard word choices to describe the experience of asthma symptoms. For example, they use a variety of affective words and phrases, such as ‘helpless’ and ‘afraid of dying’, as well as sensory words and phrases, such as ‘pressure in chest’, ‘tightness’ and ‘lungs feel closed’ to describe living with asthma (Wallace-Farquharson et al., 2022).

Experiences of living with asthma can also be accessed by examining how children live with asthma in everyday life. Specifically, children manage their asthma and minimise its effects on their everyday lives using various strategies, such as assimilating their behaviour, finding a balance and assuming control. Indeed, children wish to participate and be included in everyday activities, events and relationships without being impeded by their asthma (Hughes et al. 2018).

However, on occasion, asthma can cause children to be excluded from normal life. These children live their everyday lives with an intrusive disease, with supporters to help them and with the desire to be included in normal life (Sundal and Lykkeslet, 2020). Furthermore, they live with an awareness of their symptoms, the triggers of asthma, the appropriate use of medication and the management of their well-being. Often, these children do not like to miss school, as they feel this to be disruptive both socially and with regard to learning continuity (Searle et al., 2017). Finally, children with asthma tend to have a lower quality of everyday life in terms of physical, emotional and academic performance (Kouzegaran et al., 2018).

Although previous studies have focused on the symptoms experienced by those living with asthma and how they live their everyday lives with asthma, the present study aims to deepen and refine the understanding of the participation of children with asthma in everyday activities, and to elaborate on their descriptions of asthma symptoms when participating in everyday activities. Consequently, the purpose of this study is to explore the experiences of participating in everyday activities among children with asthma.

## Methods

The study employed a qualitative design to examine the children’s experiences, and this design was embedded in a hermeneutic phenomenological approach with lived experience, thus emphasising children’s unique experiences. The studied phenomenon was the participation in everyday activities of children with asthma (van Manen, 1990, 2014, 2015). The hermeneutic approach was central to the understanding and interpretation of the lived experiences of these children (Gadamer, 2006).

### Recruitment and collection

The data were collected through qualitative, individual, in-depth interviews to obtain the children’s experiences of participating in everyday activities (Brinkmann and Kvale, 2015). Four children with asthma aged 9–12 years (two boys and two girls) were interviewed three times, each over a period of at least 6 months to deepen the understanding of their experiences. They were interviewed at paediatric outpatient clinics; however, one child wanted the last interview to be conducted at school. The interviews lasted from 20 to 45 minutes and followed a semi-structured interview guide. The interview topic in the first and second interviews was to tell about participating in everyday activities with asthma at home, during leisure time and at school. In the third interview, the researchers read a written summary, and the children were asked to confirm and add new experiences. The children decided whether their parents were involved in the interview situation or not; one child wished to have her mother with her at the beginning of the first interview.

The inclusion criteria for the study were as follows: attending a follow-up at a paediatric outpatient clinic for asthma as a disease, and children aged 8–12 years old. The four children were recruited at two Norwegian paediatric outpatient clinics in the same area where they had regular contact and received follow-ups. The nurses at the paediatric outpatient clinics informed and recruited parents of children with asthma to the study. The parents consented to participate in the study on behalf of their children, informed the children about the study and obtained their willingness to participate. The study was conducted in a rural setting, and, thus, for practical reasons, the available children who could participate in the study during the 6-month recruitment period determined the number of participants. The author and an experienced researcher conducted the interviews with two children each; these interviews were recorded as an audio file and were transcribed verbatim by both researchers.

### Analysis

The analysis performed by the author was based on Max van Manen’s (van Manen, 1990, 2014, 2015) thematic analysis method, including his holistic, selective and detailed approaches. Firstly, the overall meaning was captured by reading each interview transcript several times. This involved identifying phrases like ‘heavy breathing’. The text was then selectively read several times to identify themes from key phrases that highlighted the phenomenon under study, such as ‘heavy breathing during physical activity’. Finally, the sentences and phrases were read in detail line by line, named, and grouped into themes. An example is as follows: ‘I get tired quickly, all the time, get more tired if I do activities, like playing football’. Overall, the analysis yielded two themes and six subthemes.

### Ethical considerations

In this study, the children’s first-hand experiences were crucial for gaining insight into their situation (Øverlien, 2013). This study was approved by the Norwegian Social Science Data Service (reference: 40237/2/JSL, 12.11.2014). Children, and especially children who are sick, are considered a vulnerable group in terms of research ethics, and they require special attention from the researcher in interview situations. Therefore, emphasis was placed on paying attention to the children’s reactions in the interview situation when talking about their experiences with their illness. Additionally, after each interview, a nurse at the outpatient clinic was available for conversation if necessary. The children did not require follow-up after the interview.

In accordance with the Declaration of Helsinki (The-Norwegian-National-Research-Ethics, 2013), written informed consent was obtained from the parents of the children by the nurse. The parents consented on behalf of their children. The children received age-appropriate information and were informed of their parents’ right to gain insights into the interviews. During the interview situation with the child, the interviewer repeated the information and the child’s rights. It was important that the children wanted to participate and knew that they were free to withdraw from the study at any time without giving any reason. No incentives were given to the participants, and the author received a salary from the University College during the study.

## Results

The findings of this study are based on interviews conducted with four children aged 9–12 years old, of whom two were girls and two were boys. Three of them lived with both parents, and one with their mother. Each child was interviewed three times. Based on these interviews, the analysis identified two themes and six sub-themes. The first theme was ‘Heavy breathing stops the body in motion’, and the second theme was ‘Help in everyday life and support to participate’.

### Heavy breathing stops the body in motion

The children with asthma who participated in this study engaged in physical activities in their everyday lives at school and in their spare time, and they reported becoming short of breath and having to stop to calm their breathing during these activities. These children also reported on occasion having to refrain from participating in activities or being absent from school due to their asthma. They had experienced asthma throughout their lives and, thus, did not have experience of not having asthma.

#### Experiencing intrusive wheezing

All the children experienced that doing physical activities at school and in their spare time that involved running made them short of breath. The children could not predict when and how short of breath they would become, but they had much experience with varying degrees of shortness of breath, ranging from slightly heavy breathing to experiencing an asthma attack that required admission to the hospital.

According to the children, situations that particularly contributed to shortness of breath were experiencing allergic reactions, receiving an allergy vaccination, or performing difficult physical training. Allergic reactions were reported to occur in response to pollen during the pollen season, contact with house dust or animals, or after eating something they were allergic to. The children could become short of breath without physical activity when they had allergic reactions. Furthermore, as described by the children, allergic reactions could worsen their shortness of breath when they were physically active.

However, they also reported becoming short of breath without allergic reactions during demanding physical activities, such as football, and especially when warming up before a football match. In this context, the children provided the following examples:

The first child said, ‘I get tired quickly, all the time. I get more tired if I do activities like playing football. It’s not fun to be tired. It becomes difficult to breathe’.

The second child expressed, ‘You lag a little behind when running. In May, it is usually the worst time [pollen]. In football, when we have hard warm-ups, I get very short of breath. I am a goalkeeper’.

The third child pointed out, ‘It hurts to breathe. If I eat something I do not tolerate, my breathing hurts. I can hardly breathe’.

The fourth child described, ‘I was breathing very heavily’.

The children also conveyed that being short of breath caused them pain. For example, they reported that it was exhausting, they got a headache, and it made them tired.

#### Calming one’s breath to participate

According to the children, when they experienced becoming short of breath during physical activity, they would calm their breathing by slowing down, stopping completely, or sitting down. They also reported sometimes having to calm themselves so as not to panic due to being short of breath and experiencing discomfort. Occasionally, they were dependent on help if an asthma attack occurred, but mostly the children reported taking care of themselves by preventing a full attack, stopping their activity and sitting down to calm their breathing. In this context, the children provided the following examples:

The first child expressed, ‘If I’m going to school and I’m in a bit of a hurry and must run and get tired, then I must slow down’.

The second child said, ‘I sit down, sit a little and breathe, and wait until I get less breathless. It takes only about 30 seconds’.

The fourth child described, ‘It is important to keep calm; just don’t panic and my mom can do what she must do, and I don’t get an asthma attack’.

#### Being sick and refraining from participating

All the children felt not only the illness in their bodies, especially when they were short of breath, but also through feeling tired and experiencing headaches. These experiences determined whether they chose to refrain from going to school or participating in leisure activities such as football. The children conveyed that they have a life that is different from that of other children, as they must listen to their bodies and constantly assess what activities they should participate in or refrain from. The children also reported that some periods are better than others, but that it is difficult to predict when bad periods, days or situations may occur. In this context, the children provided the following information:

The second child described, ‘I can’t be active all the time; I must be less active sometimes. Today, I’m going to the match, but if I don’t feel better when I get home, I don’t think I’m going to the match today. I decide for myself whether I’m tired and lethargic and cannot participate. Last week I was at home because I had a headache (due to allergies and heavy breathing)’.

The third child expressed, ‘I manage myself [and] know when to take part in activities or not. I don’t know what it’s like to have a normal life like some people have. People do not consider that some have diseases [that] can kill’.

The fourth child said: ‘Gym is difficult because of running’.

### Help in everyday life and support to participate

Having asthma made it necessary for the children to take daily asthma medicine, as well as extra medicine in some situations. All the children needed to be understood and met as sick at home, at school and during leisure activities. Indeed, the children had adapted their leisure activities in line with their needs.

#### Taking the necessary asthma medication

There was variation in terms of whether the children took their asthma medication every morning and evening, even if they expressed that it had been recommended by the doctor. Some of the children described forgetting to take their medicine, whereas some managed it completely on their own, and others managed it with some reminders from their parents. For those who forgot to take their medicine, part of the reason for this was that they did not feel a clear difference between taking the medicine and not taking it. The oldest child, who was independent in taking medicine, had a built-in routine for taking the medication every day. Moreover, the medication that they most commonly forgot to bring with them and use was the extra medication, which is taken preventively before challenging physical activity or as necessary when they become short of breath due to an allergic reaction or physical activity. The children underlined this point as follows:

The first child expressed, ‘No one helps me remember my asthma medicine. I must take my asthma medicine morning and night, but I often forget to take it. I forget six times a week. I don’t feel the difference when using asthma medicine or not because I forget to take it. I must take it regularly, and I never do that. I have started to do it. Then I forgot it again, and I started again’.

The second child said, ‘I have the medicine in the bathroom in a drawer, where I have all my things. It’s easy to take medicine. When I was smaller, my parents were with me in the bathroom and helped me’.

The third child described, ‘I take asthma medicine in the morning and evening. I manage the medicine myself but need some reminders’.

#### Being understood and met as sick

The children’s experiences of how they were met and understood as being sick with asthma varied. Specifically, some of them felt that other children and adults did not always understand what it was like to have asthma and to be short of breath, but they also had experiences of the opposite, where other people genuinely understood and considered them when they were sick. In some situations, the children felt misunderstood in the context of school or leisure activities, whereas in others they felt understood and looked after. The children attempted to master and cope with these situations and to be understood in their own ways. For instance, two children stated the following:

The second child described, ‘I don’t think they [in the class] understand what it means to have asthma. I told the teacher about the pollen allergy and got a seat furthest from the window. It’s only mum and dad who care a lot’.

The third child expressed, ‘Sometimes I get teased for it because I can’t run. They call me lazy; not everyone understands. One in the class, he understands that I get heavy breathing when he runs after me, and we play catch with each other. He doesn’t take me, but someone else, and then people click. A boy in the school says I’m a coward because I almost lose my breath when we are trying to catch each other. Dad doesn’t understand my asthma’.

#### Adapting one’s leisure activities

All the children reported not being able to participate in all leisure activities but trying to participate when they could. Indeed, the interviewed children engaged in several varied leisure activities, including physical ones, and they expressed great pleasure in participating in them. The children also participated in activities that were not physically strenuous, such as singing in a choir, playing the piano and being a football goalkeeper. The children also described finding alternative solutions for leisure activities, such as having an allergy-friendly dog due to being allergic to animals with fur and finding alternative holidays and trips (e.g. sailing trips in places without much pollen and trips to the country further south where their allergies were less severe). The children stated the following in this context:

The first child described, ‘I like jumping on the trampoline, singing in a choir, and playing football. We usually go sailing but are almost never in the forest because I’m allergic to pollen’.

The second child pointed out, ‘I play piano, which is fun, and go to acrobatics. I’m a goalkeeper and didn’t concede a single goal [in a match]. I got a dog [said enthusiastically], I am allergic to all animals with fur. But not my hypoallergenic dog’.

The third child said, ‘I like to hang out with friends and play football. It’s boring to have asthma; it stops many activities, and sometimes it can stop a sports career for those who have talent’.

The fourth child expressed, ‘We play with Barbie, ponies, teddy bears, and bouncy balls; we hang out with friends; and we jump on the trampoline’.

## Discussion

The purpose of this study was to explore the experiences of children with asthma in terms of participating in everyday activities. The findings highlighted that the interviewed children with asthma experience heavy breathing, which stops the body in motion when the wheezing is intrusive, and have to calm their breathing by stopping participation in activities. In some contexts, the children reported being too sick and, thus, having to refrain from participating in activities. According to the children, help in everyday life and support to participate in activities involve taking the necessary asthma medication, and when they were affected by asthma, it was important to be understood and met as sick. Due to their asthma, the children also often reported choosing adapted leisure activities to participate in. These findings deepen the understanding of children’s insight into having asthma when participating in everyday activities.

Previous review studies have revealed that children’s and adolescents’ descriptions of asthma symptoms are limited. They use asthma symptom terminology as well as nonstandard word choices to describe their asthma symptoms. Furthermore, children and adolescents use a variety of affective and sensory words to describe the phenomena related to asthma (Wallace-Farquharson et al., 2022) and live with an awareness of their symptoms (Searle et al., 2017). The present study elaborates on the children’s description of asthma symptoms when participating in everyday activities.

This awareness of asthma symptoms and children’s descriptions of asthma symptoms are supported by the findings of the present study, where heavy breathing was found to stop the body moving in various activities. Heavy breathing could be intrusive during activities, and the children reported having to calm their breathing by slowing down, stopping completely or sitting down in order to be able to participate in various activities, as well as sometimes having to refrain from activities altogether.

In this study, the children also reported that their asthma symptoms made them breathless even without activity, which has not been clearly described in previous studies (Hughes et al., 2018; Kouzegaran et al., 2018; Searle et al., 2017; Wallace-Farquharson et al., 2022). Moreover, in order to be able to participate, the children in this study reported choosing adapted leisure activities, which is in line with previous studies in which children have been reported to live with asthma by assimilating their behaviours to find a balance and assume control. Indeed, in this way, children can minimise the effects of asthma on their everyday lives. The results of these studies allow an understanding of young people’s behaviour when they have asthma, as they are motivated to participate and be included in everyday activities (Hughes et al., 2018).

When examining how children with asthma can be enabled to participate in activities in everyday life, their best interests must be considered. Indeed, according to Article 3 of the United Nations (UN) Convention on the Rights of the Child, the best interests of the child must be a fundamental consideration in all situations when public or private welfare organisations are responsible for following up on children, including those who have a disease such as asthma. Indeed, the protection and care necessary for the child’s well-being must always be ensured. Additionally, according to Article 13, the child must have the right to freedom of expression, which can be understood as co-determination and participation in decisions in relation to their own sickness (UNICEF).

A clear finding of this study is the children’s desire to participate in activities both at school and during their leisure time. Indeed, the children communicated these wishes and handled situations when they became short of breath or sick to enable themselves as much participation as possible; however, they depended on receiving understanding and support in situations where they were short of breath and sick. A child’s participation in everyday activities is based on the child’s best interests and the UN Convention on the Rights of the Child as an overriding principle. Therefore, ensuring these children’s protection and care to, in turn, enhance their well-being means listening to their wishes when they have difficulty breathing or are sick.

The work of philosopher Maurice Merleau-Ponty (1994) can be beneficial for further understanding the findings regarding children’s experiences of participating in everyday activities when they have asthma. Indeed, Merleau-Ponty is known as the phenomenologist of the body. According to his beliefs about the body’s existence in the world, human beings are bodily present in the world and embedded in it. Specifically, one experiences the world through one’s body, which is bodily embedded in the world, and the body inhabits two worlds: one’s own world and a shared world. Children with asthma experience the world with asthma through their bodies; they are bodily embedded in the world with asthma as a disease, with heavy breathing stopping their bodies in motion.

Furthermore, according to Merleau-Ponty (1994), the body inhabits time and space, and the contour of the body is a boundary in space. Through the body, one is able to perform actions in a room. Additionally, being in situations in time and space involves bodily encounters with situations that are not the same, and they create a life of rhythms and habits in the body. Being in the ambiguity of the world expresses itself in the ambiguity of the body and must be understood in terms of time. Therefore, the children with asthma must continuously sense their own rhythms and habits in bodily familiar actions during everyday activities in school and leisure where heavy breathing may occur, and they may experience intrusive wheezing. Although these situations and activities are repetitive and have created rhythms and habits embodied in the children’s bodies, the situations are not the same. As a result, the children learn about the likelihood and predictability of certain asthma-related experiences when, for example, they stay in larger or smaller rooms, are in a space with pollen, house dust, animals, or have eaten a certain item. Simultaneously, time is central to activities in terms of their pace; for example, the speed of the physical activities determines how strenuous they are and, thus, the likelihood of intrusive wheezing and the individual having to calm their breath to participate. The children listen to their bodies and breathing and adjust their activities according to the degree of heavy breathing; however, on occasion, they are too sick and must refrain from participating altogether.

Ultimately, according to Merleau-Ponty (1994), the body’s being in the world is characterised by a double sensation, with a shift between touching and being affected by the world. These children have gained many bodily experiences of being in the world with asthma and of the challenges of heavy breathing. The body being challenged by heavy breathing, headaches and tiredness demonstrates the body’s double sensing, where it alternates between being an active, touching body in motion and an affected body, where the activity must stop and the individual must sit or slow down due to heavy breathing. Through these experiences, the double sensation of being bodily present in the world becomes clear. In these situations of experiencing intrusive wheezing, children need help in everyday life and support to participate in activities, take the necessary asthma medicine and be understood and met as sick. Due to their many bodily experiences, with heavy breathing stopping their bodies in motion, they tend to adapt their leisure activities.

### Strengths and limitations of the study

A strength of this study was interviewing the children three times over at least half a year. This provided the opportunity to observe their development across different seasons, ensure variation in their experiences and to get to know the children well.

However, a possible limitation of this study is that there were only four children who participated, although interviewing each child three times over time may compensate for this by providing more variations in the data. The author and another researcher planned the interviews and interviewed two children each, and the fact that there were two researchers contributed to the opportunity to reflect on the interviews with the children. However, the author analysed the findings alone, which may have reduced the trustworthiness of the results. However, it was an advantage that the author has experience analysing qualitative data. Using the COREQ checklist helped to ensure the quality of the research process and the results.

## Conclusion

The purpose of this study was to explore the experiences of children with asthma in terms of participating in everyday activities. To participate in everyday activities, children with asthma require insight into and an understanding of the importance of taking asthma medication as recommended, and they require support to take it. The children must also stop their activities when dealing with situations of heavy breathing, and they must be met with understanding and help in situations in their everyday lives when they are sick. Moreover, these children must continuously listen to their bodies and breathe to balance their activity levels appropriately. This behaviour would allow these children the highest possible degree of participation in activities in the context of school and leisure in everyday life, as well as providing them with co-determination and influence in situations. It is important for nurses and clinicians to understand children’s everyday experiences of having asthma; thus, these findings represent an important contribution to the understanding of nursing care for children with asthma as part of the healthcare system. This knowledge may prevent asthma attacks and hospital admissions for children with asthma. Taken together, this study’s findings may contribute to the enhancing the quality of life of children with asthma.

## Key points for policy, practice and/or research

- Children with asthma need insight into the importance of taking asthma medicine.
- Children require follow-up to take asthma medicine, and this may prevent asthma attacks and hospital admission.
- Children listen to their bodies and breathe to balance their activity levels, and this allows the highest possible degree of participation in activities in everyday life.
